# The Non-Peptide MAS-R Agonist AVE0991 Alleviates Colitis Severity in Mice and Exhibits an Additive Effect with Azathioprine

**DOI:** 10.3390/ijms26125784

**Published:** 2025-06-17

**Authors:** Maitham A. Khajah, Sana Hawai, Ahmad Barakat

**Affiliations:** College of Pharmacy, Kuwait University, P.O. Box 24923, Safat 13110, Kuwait; sana.hawai@ku.edu.kw (S.H.); ahmad.barkat@ku.edu.kw (A.B.)

**Keywords:** colitis, DSS, AVE0991, MAS-R, azathioprine, inflammation, signal transduction

## Abstract

A growing body of evidence suggests the potent anti-inflammatory properties of the newly discovered arm of the renin–angiotensin–aldosterone system, ACE2/Ang-(1–7)/MasR, in various disease conditions. Our group was the first to report the anti-inflammatory properties of the Ang-(1–7) polypeptide in the murine dextran sulfate sodium (DSS) colitis model. Both its short half-life and high degradation rate limit the clinical use of Ang-(1–7). One way to compensate for these limitations is through the use of the non-peptide MasR agonist AVE0991. Herein, we aimed to study the anti-inflammatory effects of AVE0991 using the DSS model and the possible synergistic effects with other clinically available medications. Colitis severity was determined using both prophylactic and treatment approaches by gross anatomical and histological assessments and daily weight changes. The colonic expression level/activity of various pro-inflammatory and adhesion molecules was determined by western blotting, immunofluorescence, and proteomic profiling. We showed that AVE0991 treatment significantly reduced colitis severity more effectively with the prophylactic than the treatment approach. An additive anti-inflammatory effect was observed in the combination regimen with AVE0991 plus azathioprine, which was mediated through an increased colonic expression level of mucins and focal adhesion kinase, decreased colonic activity of p38 MAPK and Akt, and decreased colonic expression level of various pro-inflammatory mediators. In conclusion, these data suggest a promising potential for the non-peptide MasR agonist AVE0991 in the treatment of inflammatory bowel disease.

## 1. Introduction

There has been a lot of attention in recent years regarding the role of the renin–angiotensin–aldosterone system (RAAS) in disease conditions beyond what was historically known in regulating the kidney and the cardiovascular system. This peptide-based system consists of two main axes that balance and sometimes negate each other: ACE/Ang II/ AT_1_/_2_R and ACE2/Ang-(1–7)/MasR. In the kidney, renin is released by the juxtaglomerular cells and converts angiotensinogen to angiotensin I (Ang I) by the removal of the N-terminal region. The ACE/Ang II/AT_1_R axis uses angiotensin-converting enzyme (ACE) and chymase-1 to convert Ang I to angiotensin II (Ang II) by the removal of two C-terminal amino acids [1,2,3,4,5]. Ang II signals through the angiotensin type 1 (AT_1_) receptor, resulting in various downstream effects, such as enhanced cell proliferation, vasoconstriction, and increased salt and water retention [1,2,3]. Ang II also signals through the angiotensin type 2 (AT_2_) receptor to oppose the A1TR-induced effects. There are various drugs that target the RAAS, such as ACE inhibitors (ACEi), AT_1_ receptor blockers (ARBs), and renin blockers, which are currently used in the treatment of numerous diseases such as hypertension, chronic heart failure, chronic kidney disease, and myocardial infarction [4].

The alternative ACE2/Ang-(1–7)/MasR axis produces angiotensin 1–7 (Ang-(1–7) directly from either Ang I or Ang II and indirectly from Ang I. The direct hydrolysis converts Ang II to Ang-(1–7) by ACE2 predominantly, as well as by carboxypeptidase (PCP) and prolylendopeptidase (PEP). Furthermore, Ang I can be directly converted to Ang-(1–7) by PEP, neutral endopeptidase (NEP), neprilysin, and thimet oligopeptidase. The indirect hydrolysis consists of a two-step process, Ang I is converted to angiotensin-(1–9) (Ang-(1–9) by ACE2 which is then converted to Ang-(1–7) by ACE or minimally by NEP. ACE2 is required for both pathways, but the catalytic activity is higher with Ang II than with Ang I. Ang-(1–7) signaling occurs through the G-protein-coupled transmembrane mitochondrial assembly receptor (MasR). Ang-(1–7) effects (which oppose Ang II effects) include dilation of blood vessels, as well as reduction in cell proliferation and water/salt retention. Some reports have shown that Ang-(1–7) level is elevated during long-term blockage of the RAAS using ACEI or ARBs, suggesting the importance of the ACE2/Ang-(1–7)/MasR axis in the therapeutic effects of these drugs [2,6,7,8,9]. Since Ang-(1–7) is a peptide hormone that can be broken down by numerous proteolytic enzymes, this poses a huge limitation for its clinical utility in the treatment of various disease conditions. It should be noted that there is recent evidence regarding the role of melanocortin and its receptors in IBD pathogenesis and their possible interaction with the RAAS [10,11].

AVE0991 is a non-peptide MasR agonist, which is not affected by the proteolytic enzymes and produces similar effects to Ang-(1–7) in various organs, including the kidneys, blood vessels, and heart. It also has a relatively longer half-life than Ang-(1–7) and specificity for the MasR [6,8,9,12]. For example, AVE0991 treatment induced vasodilation in the isolated aortic ring from mice, which was abolished in MasR-deficient mice [4]. AVE0991 and Ang-(1–7) compete on similar binding sites for the induction of nitric oxide (NO) release using the bovine aortic endothelial cells. In fact, the level of NO release was greater with AVE0991 than Ang-(1–7), and the binding of Ang-(1–7) to MasR was displaced by AVE0991 in the Chinese hamster ovary cells [4]. These data suggest that this non-peptide compound can be used as an alternative to Ang-(1–7) in the clinical setting to compensate for its limitations in terms of its very high chance of degradation and short half-life.

There is an emerging body of evidence suggesting an important role for Ang 1-7/AVE0991 in various disease conditions including acute kidney injury [4], erectile dysfunction [8], cardiovascular diseases [13], atherosclerosis [14], preeclampsia [7], Alzheimer’s disease [15], chronic asthma and acute respiratory distress syndrome [16], and rheumatoid arthritis [17].

Inflammatory bowel disease (IBD) is a chronic pathological condition affecting the gastrointestinal tract and comprises two main conditions: Crohn’s disease and ulcerative colitis [18,19,20,21]. From various reports, Ang II has been shown to play an important role in colitis pathogenesis. For example, reducing Ang II levels through treatment with ACEI or ARBs resulted in significant improvement in colitis severity in chemically induced colitis models such as 2, 4, 6-trinitrobenzene sulfonic acid (TNBS) and dextran sulfate sodium (DSS) [22,23,24,25,26,27,28]. Also, reduced colitis severity was evident in mice deficient in angiotensin [28] or the ATR1a gene [29]. In the clinical setting, ACE gene polymorphism was detected in IBD patients and is associated with reduced ACE serum levels; this might be associated with the disease pathogenesis and its extra-intestinal side effects [30,31,32]. Regarding Ang-(1–7), ACE2 expression has been found in epithelial and sub-mucosal cells throughout the GIT with significant expression in the ileum and the colon [33,34,35]. In addition, enhanced expression of ACE2 mRNA levels has also been also found in patients with IBD [36]. In fact, our group was the first to demonstrate direct anti-inflammatory properties of Ang-(1–7) using a murine DSS colitis model [5,37]. These data suggest a promising role for Ang-(1–7) in the treatment of IBD when taking into consideration the drawbacks of the currently available treatment options in terms of side effects profile, resistance [38], and limited efficacy [39]. Therefore, there is a need to define new therapeutic targets that may offer alternatives to treat this chronic inflammatory condition and provide a cure. The main drawback of using Ang-(1–7) in the clinical setting is its short half-life and degradation issue (since it is a polypeptide). Therefore, using a non-peptide agonist of the MasR such as AVE0991 might solve this issue. To our knowledge, there are no published reports regarding the role of AVE0991 in animal models of IBD.

In this report, we aimed to determine if AVE0991 can modulate colitis severity using the murine DSS model with various approaches (prophylactic and treatment), and its effect in combination regimens with other known treatments. We demonstrated proof-of-concept for the anti-inflammatory properties of AVE0991 in reducing colitis severity in mice. We showed that AVE0991 treatment significantly reduced colitis severity with the prophylactic and treatment (on an established colitis setting) approaches. Single administration of AVE0991 was as effective as daily administration with the prophylactic approach. Also, higher doses of AVE0991 were required to reduce colitis severity with the treatment compared to the prophylactic approach. An additive anti-inflammatory effect was observed in the combination regimen with AVE0991 (30 mg/kg) plus azathioprine, which was mediated through increased colonic expression levels of mucins and focal adhesion kinase, decreased colonic activity of p38 MAPK and Akt, and decreased colonic expression levels of various pro-inflammatory mediators. These data suggest a promising potential for the non-peptide MasR agonist AVE0991 in the treatment of IBD.

## 2. Results 

### 2.1. Effect of AVE0991 on Colitis Severity Using the Prophylactic Approach

**Figure 1:**
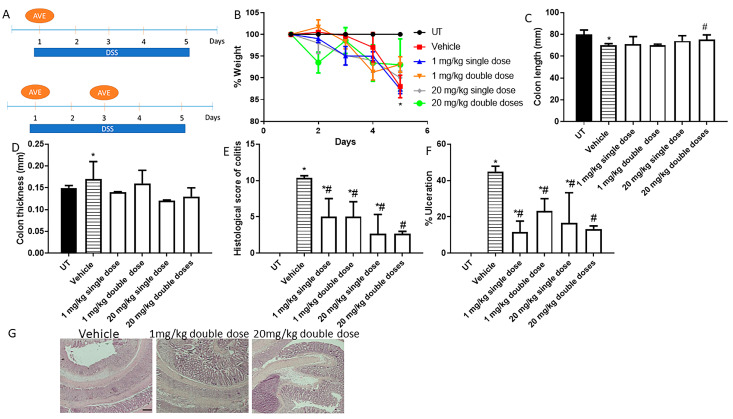
Effect of administration of a single or double dose of AVE0991 on colitis severity using the prophylactic approach. (**A**) shows the protocols used in this part of the study. (**B**) shows percentage body weight changes in vehicle- or AVE0991-treated mice after DSS administration compared to untreated (UT) mice receiving tap water only. Colon length (**C**) and thickness (**D**) were determined in vehicle- (hatched bars) or AVE0991-treated mice (open bars) compared to the UT group (solid bars). (**E**,**F**) represent the histological assessment of colitis severity and the percentage of ulceration in the whole colon section, respectively, in the indicated groups. (**G**) illustrates a colon section taken from a mouse treated by daily i.p vehicle plus DSS, where there is significant mucosal destruction, goblet cell depletion, muscle thickness, and immune cells recruitment, or a mouse treated with 1 or 20 mg/kg double dose of AVE0991, with little improvement in colitis severity. * denotes a significant difference from UT mice, with *p* < 0.05. # denotes a significant difference from DSS/i.p vehicle-treated mice, with *p* < 0.05 (n = 3 per group). The scale bar represents 150 µm.

The effect of AVE0991 on modulating colitis severity in mice was tested through various prophylactic approaches, such as single i.p injection on day 1, two i.p injections on days 1 and 3, and daily i.p injections on days 1–5. Figure 1A shows the protocol used for the single and double doses of AVE0991. As shown in Figure 1B, DSS/i.p vehicle administration resulted in approximately 13% drop in body weight on day 5 compared to the UT (healthy mice receiving tap water only) group. Single- or double-dose administration of AVE0991 at 1 or 20 mg/kg did not recover the drop in body weight to the level of the UT group. DSS/i.p vehicle administration resulted in a decrease in colon length (Figure 1C) and an increase in colon thickness (Figure 1D) compared to the UT group, this was also not prevented with single or double doses of AVE0991 administration. As indicated in Table 1, at the gross level, DSS administration for 5 days with daily i.p injections of vehicle resulted in edema (66%), diarrhea (66%), blood in stool (66%), and adhesions (66%), none of which was seen in the UT group. Treatment with single or double doses of AVE0991 at 1 or 20 mg/kg resulted in the absence of these parameters of colitis severity at the gross level. As shown in Figure 1E, DSS/i.p vehicle treatment resulted in a significant increase in the histological score of colitis and the percentage of ulceration involved in the whole colon (Figure 1F). Treatment with AVE0991 at single or double doses resulted in a 50–60% reduction in the histological score of colitis severity and percentage of ulceration. Figure 1G shows H/E sections from mice treated with DSS/I.p vehicle which demonstrates significant destruction of mucosal architecture, goblet cell depletion, ulceration, and immune cell recruitment. Sections taken from mice treated with a double dose of 1 or 20 mg/kg show partial resolution of these parameters.

**Figure 2:**
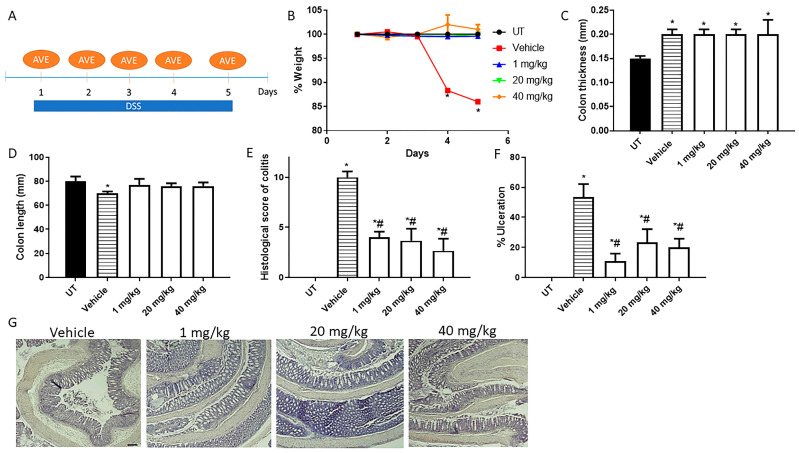
Effect of daily administration of AVE0991 on colitis severity using the prophylactic approach. (**A**) shows the protocol used in this part of the study. (**B**) shows percentage body weight changes in vehicle- or AVE0991-treated mice after DSS administration compared to UT mice. Colon length (**C**) and thickness (**D**) were determined in vehicle- (hatched bars) or AVE0991-treated mice (open bars) compared to the UT group (solid bars). (**E**,**F**) represent the histological assessment of colitis severity and the percentage of ulceration in the whole colon section, respectively, in the indicated groups. (**G**) illustrates a colon section taken from a mouse treated by daily i.p vehicle plus DSS, or a mouse treated with 1, 20, or 40 mg/kg daily dose of AVE0991 with significant improvement in colitis severity. * denotes a significant difference from UT mice, with *p* < 0.05. # denotes a significant difference from DSS/i.p vehicle-treated mice, with *p* < 0.05 (n = 3 per group). The scale bar represents 150 µm.

Next, we determined the effect of daily i.p administration with AVE0991 on colitis severity using the prophylactic approach. Figure 2A shows the protocol used for daily i.p with AVE0991. As shown in Figure 2B, DSS/i.p vehicle administration resulted in approximately a 15% drop in body weight on day 5 compared to the UT group. Daily i.p with AVE0991 at doses of 1, 20, and 40 mg/kg restored the drop in body weight to the UT level. Colon thickness was significantly increased in DSS-treated mice either with vehicle or various doses of AVE0991 compared to UT mice (Figure 2C). In contrast, colon length was restored to UT level in response to AVE0991 (Figure 2D). As indicated in Table 2, DSS/i.p vehicle resulted in edema (100%), diarrhea (66%), blood in stool (66%), and adhesions (100%). Daily AVE0991 at 1, 20, and 40 mg/kg resulted in the absence of these parameters of colitis severity at the gross level. As shown in Figure 2E,F, daily AVE0991 significantly reduced the histological score of colitis severity and percentage of ulceration at all doses used. Histological examination (Figure 2G) showed that daily AVE0991 significantly decreased mucosal damage, ulceration, goblet cell depletion, and the degree of immune cell recruitment to the colon when compared to the DSS/i.p vehicle group.

### 2.2. Effect of AVE0991 on Colitis Severity Using the Treatment Approach

**Figure 3:**
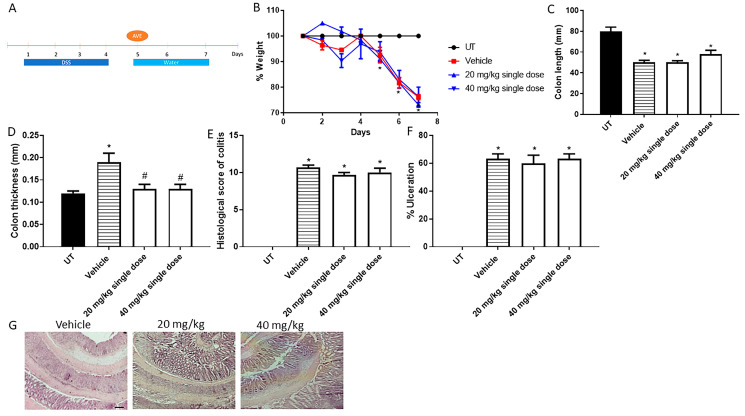
Effect of administration of a single dose of AVE0991 on colitis severity using the treatment approach. (**A**) shows the protocol used in this part of the study. (**B**) shows percentage body weight changes in vehicle- or AVE0991-treated mice after DSS administration compared to UT mice. Colon length (**C**) and thickness (**D**) were determined in vehicle- (hatched bars) or AVE0991-treated mice (open bars) compared to the UT group (solid bars). (**E**,**F**) represent the histological assessment of colitis severity and the percentage of ulceration in the whole colon section, respectively, in the indicated groups. (**G**) illustrates a colon section taken from a mouse treated by daily i.p vehicle plus DSS, or a mouse treated with a single dose of AVE0991 (20 or 40 mg/kg) with little improvement in colitis severity. * denotes a significant difference from UT mice, with *p* < 0.05. # denotes a significant difference from DSS/i.p vehicle-treated mice, with *p* < 0.05 (n = 3 per group). The scale bar represents 150 µm.

The effect of AVE0991 on colitis severity was also examined using the treatment approach (i.e., after colitis induction) with a single dose (at day 5) or daily doses (at days 5, 6, and 7) regimens. Figure 3A shows the protocol used for the single-treatment approach. As shown in Figure 3B, around a 30% drop in body weight from baseline levels was seen with DSS/i.p vehicle administration and with the single dose regimen of AVE0991 at 20 and 40 mg/kg. AVE0991 reversed the increase in colon thickness but not the decrease in colon length induced by DSS (Figure 3C,D). There was an improvement in some of the gross parameters of colitis severity such as diarrhea and blood in stool only with the 40 mg/kg dose of AVE0991 (Table 3). At the histological level, single AVE0991 treatment did not improve the score of colitis severity or percentage of ulceration (Figure 3E,F), which is also reflected in the selected H/E slides provided in Figure 3G.

**Figure 4:**
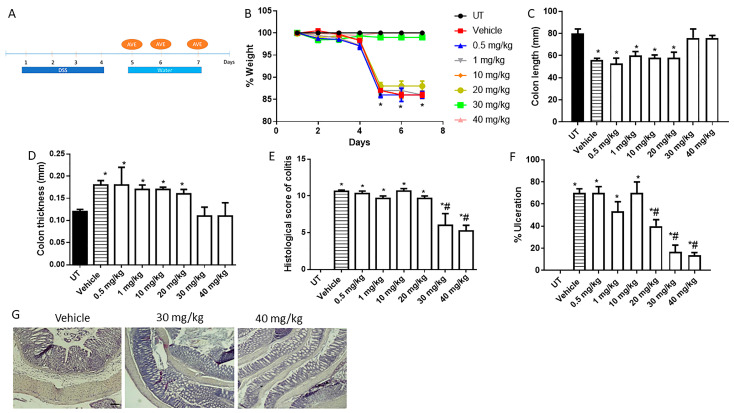
Effect of daily administration of AVE0991 on colitis severity using the treatment approach. (**A**) shows the protocol used in this part of the study. (**B**) shows percentage body weight changes in vehicle- or AVE0991-treated mice after DSS administration compared to UT mice. Colon length (**C**) and thickness (**D**) were determined in vehicle- (hatched bars) or AVE0991-treated mice (open bars) compared to the UT group (solid bars). (**E**,**F**) represent the histological assessment of colitis severity and the percentage of ulceration in the whole colon section, respectively, in the indicated groups. (**G**) illustrates a colon section taken from a mouse treated by daily i.p vehicle plus DSS, or a mouse treated with 30 or 40 mg/kg daily dose of AVE0991 with significant improvement in colitis severity. * denotes a significant difference from UT mice, with *p* < 0.05. # denotes a significant difference from DSS/i.p vehicle-treated mice, with *p* < 0.05 (vehicle n = 6, UT and the treatment groups n = 4 per group). The scale bar represents 150 µm.

Next, the daily AVE0991 regimen was examined with the treatment approach, as shown in Figure 4A. Recovery from DSS-induced weight loss, decrease in colon length, increase in colon thickness, and gross colitis parameters was seen with daily AVE0991 treatment at 30–40 mg/kg (Figure 4B–D, and Table 4). Also, the histological score of colitis severity and percentage of ulceration were significantly decreased with 30–40 mg/kg AVE0991 (Figure 4E,F). As shown in the H/E stain of colon sections presented in Figure 4G, there was an improvement in the degree of mucosal damage and ulceration with 30–40 mg/kg AVE0991 daily treatment.

### 2.3. Effect of Methylprednisolone, Budesonide, and Azathioprine; Either as Monotherapy or in Combination with AVE0991 on Colitis Severity Using the Treatment Approach

**Figure 5:**
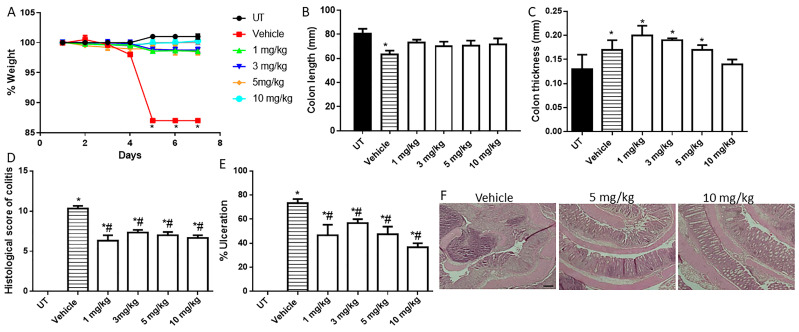
Effect of daily administration of azathioprine on colitis severity using the treatment approach. (**A**) shows percentage body weight changes in vehicle- or azathioprine-treated mice after DSS administration compared to UT mice. Colon length (**B**) and thickness (**C**) were determined in vehicle- (hatched bars) or azathioprine-treated mice (open bars) compared to the UT group (solid bars). (**D**,**E**) represent the histological assessment of colitis severity and the percentage of ulceration in the whole colon section, respectively, in the indicated groups. (**F**) illustrates a colon section taken from a mouse treated by daily i.p vehicle plus DSS, or a mouse treated with 5 or 10 mg/kg daily dose of azathioprine with some improvement in colitis severity. * denotes a significant difference from UT mice, with *p* < 0.05. # denotes a significant difference from DSS/i.p vehicle-treated mice, with *p* < 0.05 (vehicle n = 4, UT and the treatment groups n = 3 per group). The scale bar represents 150 µm.

We previously conducted dose-response studies using the DSS colitis model with the treatment approach with methylprednisolone and budesonide [40,41], and we chose a suboptimal dose of both drugs (methylprednisolone 5 mg/kg, and budesonide 0.5 µg/kg) to be used in a combination regimen with AVE0991 in the current study. We first performed a dose-response daily treatment approach with azathioprine to choose a suboptimal dose to be used in combination with AVE0991. As shown in Figure 5 and Table 5, all doses of azathioprine used (1, 3, 5, and 10 mg/kg) showed roughly similar responses in reducing colitis severity at gross and histological levels. Therefore, we decided to use the 1 mg/kg dose of azathioprine in the combination regimen with AVE0991.

**Figure 6:**
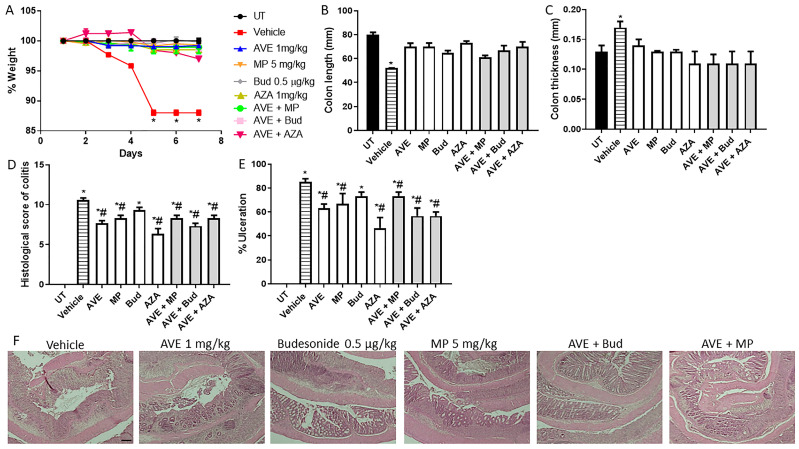
Effect of methylprednisolone, budesonide, and azathioprine; either as monotherapy or in combination with AVE0991 (1 mg/kg dose) on colitis severity using the treatment approach. (**A**) shows percentage body weight changes in the different treatment approaches. Colon length (**B**) and thickness (**C**) were determined in the vehicle (hatched bars) or different treatment approaches (open bars for monotherapy and gray bars for combination regimens) compared to the UT group (solid bars). (**D**,**E**) represent the histological assessment of colitis severity and the percentage of ulceration in the whole colon section, respectively, in the indicated groups. (**F**) illustrates a colon section taken from different treatment regimens. * denotes a significant difference from UT mice, with *p* < 0.05. # denotes a significant difference from DSS/i.p vehicle-treated mice, with *p* < 0.05 (n = 3 per group). The scale bar represents 150 µm.

**Figure 7:**
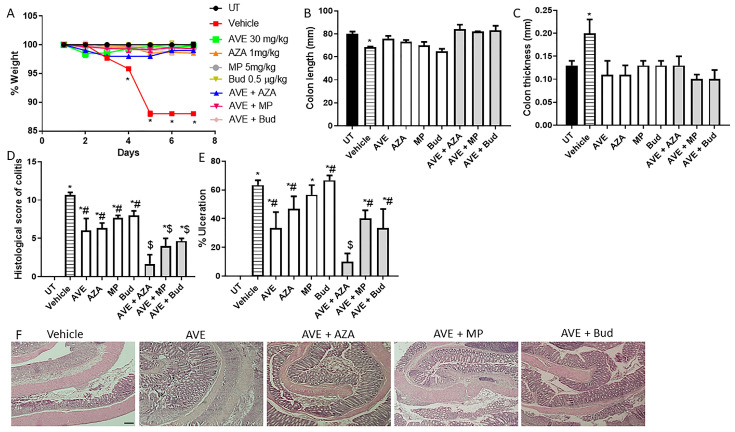
Effect of methylprednisolone, budesonide, and azathioprine; either as monotherapy or in combination with AVE0991 (30 mg/kg dose) on colitis severity using the treatment approach. (**A**) shows percentage body weight changes in the different treatment approaches. Colon length (**B**) and thickness (**C**) were determined in the vehicle (hatched bars) or different treatment approaches (open bars for monotherapy and gray bars for combination regimens) compared to the UT group (solid bars). (**D**,**E**) represent the histological assessment of colitis severity and the percentage of ulceration in the whole colon section, respectively, in the indicated groups. (**F**) illustrates a colon section taken from different treatment regimens. * denotes a significant difference from UT mice, with *p* < 0.05. # denotes a significant difference from DSS/i.p vehicle-treated mice, with *p* < 0.05. $ denotes a significant difference from monotherapy, with *p* < 0.05 (n = 3 per group). The scale bar represents 150 µm.

A combination regimen using 1 mg/kg AVE0991 (Figure 6 and Table 6) did not induce an additive effect, but a combination regimen using 30 mg/kg AVE0991 (Figure 7 and Table 7) induced an additive anti-inflammatory effect with all the tested drugs, with the best response observed with the AVE 0991 plus azathioprine combination regimen. Regarding the effect of the combination of AVE 0991 plus azathioprine at the histological score of colitis and percentage of ulceration (Figure 7D,E), monotherapy resulted in approximately a 40% reduction in these parameters, compared to an 80% reduction with the combination regimen.

### 2.4. Effect of AVE0991 Plus Azathioprine Combination Regimen on the Colonic Expression of Adhesion Molecules

**Figure 8:**
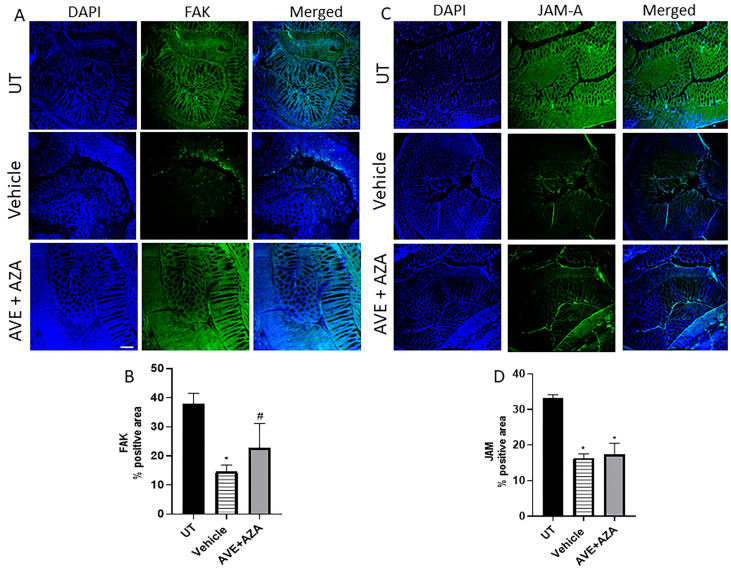
Immunofluorescent detection of FAK and JAM-A in colon sections. Colon sections taken from UT mice or mice treated with DSS plus AVE0991 (30 mg/kg) and azathioprine (1 mg/kg) combination or vehicle were immunostained with antisera against focal adhesion kinase (FAK, (**A**)) or junctional adhesion molecule-A (JAM-A, (**C**)). (**B**,**D**) represent the quantitative assessment of fluorescence intensity (arbitrary units). Histobars represent means ± SEM for 3 mice in each group. * denotes a significant difference from UT mice, with *p* < 0.05; # denotes a significant difference from DSS/i.p vehicle-treated mice, with *p* < 0.05. The scale bar represents 150 µm.

The expression profile of focal adhesion kinase (FAK) and junctional adhesion molecule (JAM) was determined in the colonic section using IF analysis. As shown in Figure 8, there was a high expression level of FAK and JAM in the UT (healthy) group. DSS/i.p administration significantly decreased their expression profile, which was recovered with the AVE plus azathioprine combination regimen in the case of FAK (but not JAM).

### 2.5. Effect of Methylprednisolone, Budesonide, and Azathioprine; Either as Monotherapy or in Combination with AVE0991 on Colonic Mucus Levels

**Figure 9:**
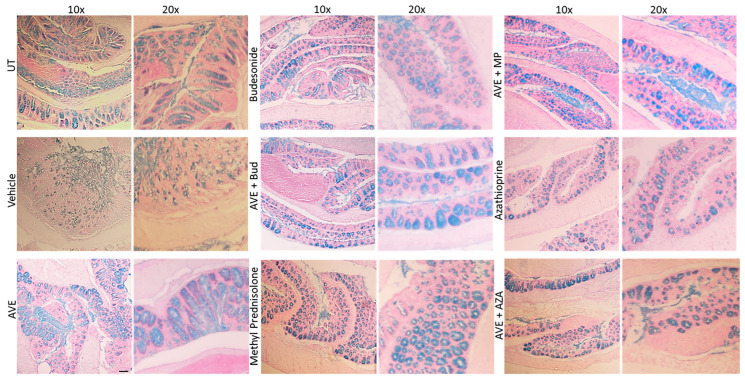
Effect of methylprednisolone, budesonide, and azathioprine; either as monotherapy or in combination with AVE0991 (30 mg/kg dose) on colonic mucus levels Colon sections taken from UT mice or mice treated with different treatment approaches, as monotherapy or combination regimens, were stained with alcian blue stain. The sections represent a sample from three separate experiments. The scale bar represents 150 µm.

The expression profile of mucus was significantly decreased with DSS administration compared to controls. Treatment with monotherapy or combination regimens resulted in increased mucus expression similar to control levels (Figure 9).

### 2.6. Effect of AVE0991 Plus Azathioprine Combination Regimen on the Colonic Phosphorylation Level of Pro-Inflammatory Signaling Molecules

**Figure 10:**
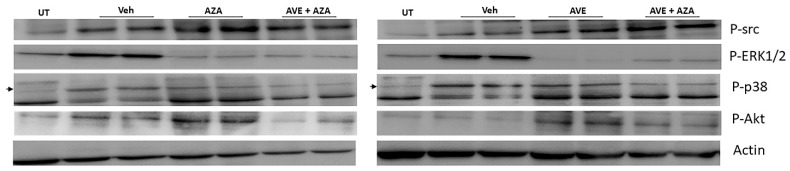
Western blot analysis of the phosphorylated levels of src, ERK1/2, p38 MAPK, and Akt in colon sections. Colon sections taken from UT mice or mice treated with DSS plus AVE0991 (30 mg/kg), azathioprine (1 mg/kg), a combination regimen, or vehicle were used for western blot analysis. The blots represent a sample from three separate experiments.

The phosphorylated levels of src, ERK1/2, p38 MAPK, and Akt were determined in the colonic sections of UT, monotherapy with AVE or azathioprine, or a combination regimen, using western blotting. As shown in Figure 10, DSS/i.p vehicle administration increased the phosphorylation level of src, ERK1/2, p38 MAPK, and Akt. P-ERK1/2, (but not P-src), was reduced by the various treatment approaches. The combination regiment reduced P-p38 and P-Akt more than monotherapy with either AVE0991 or azathioprine.

### 2.7. Effect of AVE0991 Plus Azathioprine Combination Regimen on the Colonic Expression Level of Various Pro-Inflammatory Molecules

**Figure 11:**
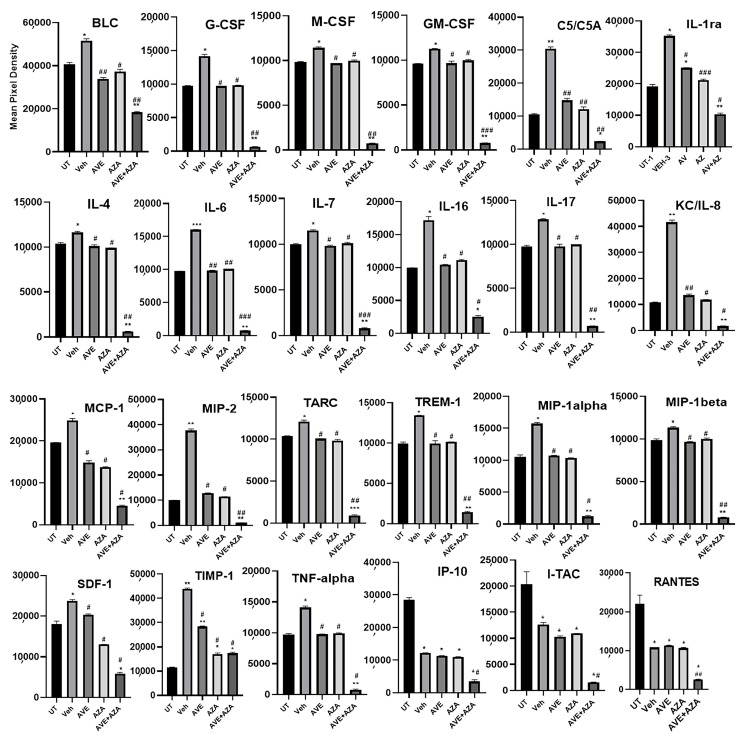

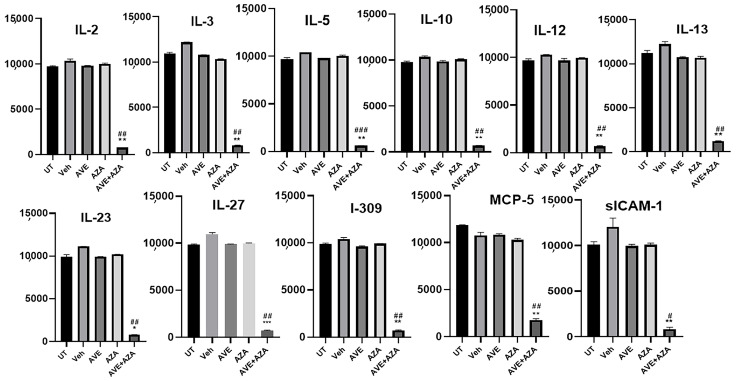
Proteomic profiling of various pro-inflammatory molecules in colon sections. Colon sections taken from UT mice or mice treated with DSS plus AVE0991 (30 mg/kg), azathioprine (1 mg/kg), a combination regimen, or vehicle were used for proteomic profiling analysis. The panels show densitometric analysis of protein profiling for the various tested targets. Asterisks denote significant differences from UT mice, with *p* < 0.05 (*), *p* < 0.001 (**), or *p* < 0.0001 (***). Asterkis denotes a significant difference from DSS/i.p vehicle-treated mice, with # *p* < 0.05, ## *p* < 0.001, or ### *p* < 0.0001 (n = 4 per group).

The expression levels of various pro-inflammatory molecules were determined in the colonic sections of UT, monotherapy with AVE or azathioprine, or a combination regimen using proteomic profiling. As shown in Figure 11, the expression levels of the following molecules were significantly increased in DSS/i.p vehicle relative to the UT group, significantly downregulated with monotherapy, and more profound downregulation with a combination regimen: B-lymphocyte chemoattractant (BLC), granulocyte colony-stimulating factor (G-CSF), macrophage colony-stimulating factor (M-CSF), GM-CSF, interleukin- receptor-a (IL-1ra), IL-4, IL-6, IL-7, IL-8, IL-16, IL-17, monocyte chemoattractant protein-1 (MCP-1), macrophage inflammatory protein-1 alpha (MIP-1 alpha), MIP-1 beta, MIP-2, thymus and activation regulated chemokine (TARC), triggering receptor expressed on myeloid cells-1 (TREM-1), stromal cell-derived factor-1 (SDF-1), tissue inhibitor of metaloporeninase-1 (TIMP-1), and tumor necrosis factor-alpha (TNF-alpha).

**Figure 12:**
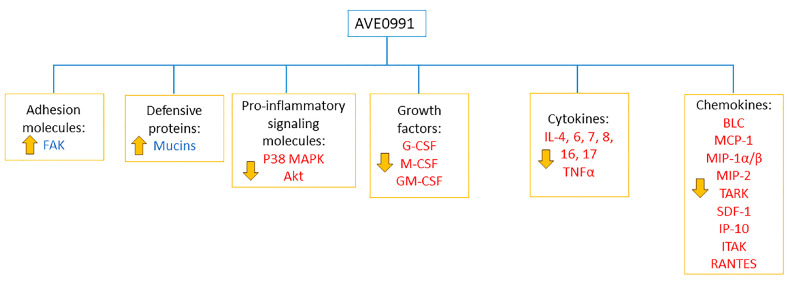
Model for AVE0991 anti-inflammatory properties. AVE0991 treatment reduced colitis severity in mice by modulating the expression/activity profile of various molecules involved in the inflammatory process.

As shown in Figure 12, the expression profile of the C-X-C motif chemokine ligand 10 (CXCL10) or (IP-10), C-X-C motif chemokine 11 (I-TAC), and CCL-5 chemokine (RANTES) were significantly decreased with DSS/i.p or monotherapy compared to the UT group, with a more profound decrease seen with the combination regimen. The expression profile of the following molecules was significantly decreased only with the combination regimen: IL-2, -3, -5, -10, 12, -13, -23, -27, CCL-1 (I-309), monocyte chemoattractant protein-5 (MCP-5), and soluble intracellular adhesion molecule-1 (sICAM-1).

## 3. Discussion

In this report, we demonstrated that treatment with the non-peptide MasR agonist AVE0991 significantly reduced colitis severity in mice when given at both prophylactic and treatment approaches. An additive anti-inflammatory effect was observed in the combination regimen with AVE0991 plus azathioprine using the treatment approach. These effects were mediated, in part, by modulating the colonic expression/activity of various molecules involved in the inflammatory response, such as adhesion molecules (FAK), mucins, downstream signaling pathways (p38 MAPK and Akt), and various pro-inflammatory cytokines and chemokines.

We previously demonstrated that daily administration of Ang 1–7 significantly decreased colitis severity at both gross and histological levels, which was mediated, in part, by decreased colonic expression/activity of various pro-inflammatory proteins, such as ERK1/2, p38 MAPK, Akt, and Ang II [5]. In another report [37], we also demonstrated that Ang-(1–7) treatment significantly decreased the colonic expression level of various proteins using a protein profiling technique, including IL-1a, IL-5, IL-6, IL-10, IL-27, ITAC, KC, and C5/C5a. The colonic expression of many of these pro-inflammatory molecules was also decreased by AVE0991 treatment, such as p38 MAPK and Akt (Figure 10), and IL-5, IL-6, IL-10, IL-27, and ITAC (Figure 11 and Figure 12).

There is a growing body of evidence suggesting the anti-inflammatory properties of AVE0991 in various disease models. AVE0991 treatment in the APP/PS1 double-transgenic mouse model of Alzheimer’s disease suppressed astrocyte-mediated inflammation by downregulating the expression profile of IL-1β, IL-6, and TNF-α in the brain cortex [42]. AVE0991 treatment also produced antihypertensive effects and alleviated vascular responses in the Ang II-induced hypertension model in rats. This was mediated, in part, by reduced cardiac inflammatory and oxidative stress activity including MMP-2 and MCP-1 [43]. Using the ovalbumin-induced acute asthmatic murine model, it was demonstrated that AVE0991 treatment significantly alleviated macrophage infiltration in the airways, in part, through downregulation of CCL2 expression and MAPK phosphorylation [44]. Furthermore, AVE0991 treatment attenuated the aging-related neuroinflammation via suppression of microglial-mediated inflammatory response by decreasing the expression levels of IL-1β, IL-6, and TNF-α in the brain [45]. Antiatherosclerosis properties were demonstrated by AVE0991 which was mediated through the inhibition of perivascular inflammation by reducing the expression of various cytokines and chemokines such as IL-1β, TNF-α, CCL2, and CXCL10 [14]. The epithelial cells play an important role in protecting the intestinal mucosa from various triggers, which may result in chronic inflammation and tissue damage. These specialized cells produce mucus from goblet cells which play an important role in health and disease, including resistance to bacterial translocation [46]. Herein, we showed that AVE0991, azathioprine, methylprednisolone, and budesonide, either as monotherapy or in a combination regimen, restored mucus levels similar to those in the healthy group.

These data, along with the data presented in this report, demonstrate a growing body of evidence for potent anti-inflammatory properties for AVE0991 in various animal models of inflammatory disorders. The anti-inflammatory properties of AVE0991 must also be examined in other animal models of colitis and in colonic tissues taken from humans with different severity of IBD (Crohn’s disease and ulcerative colitis) to confirm its efficacy. Also, examining its efficacy using other routes of administration is required. In conclusion, our data suggest a promising role for AVE0991 in the treatment of IBD, which requires further investigations. Figure 12 shows a model illustrating the potential mechanisms of the anti-inflammatory properties of AVE0991 in reducing colitis severity.

## 4. Materials and Methods

### 4.1. Animals

Female BALB/c mice (6–10 weeks old, mean weight 20 g) were supplied by the Animal Resource Center of the Health Sciences Center at Kuwait University. All animals were kept under standard conditions, including controlled temperature (25 °C) and a 12 h light-dark cycle, and had free access to food and drinking water ad libitum. All experimentations were approved by the Animal Care Committee at Kuwait University Health Sciences Center and conformed to their rules and regulations. During this study, the general health and well-being of the mice were continuously monitored and overseen by a veterinary physician in the animal house. All mice were monitored for their eating/drinking habits, activity, or other severe signs of hunched or lateral recumbency, starry fur, or lethargy. There were no signs of illn Caess or mortality during the treatment time points that required sacrificing the animals. All experiments were performed in a blinded manner using specific letter codes.

### 4.2. Induction of Colitis

Colitis was induced in mice by delivering DSS polymers (3.5% *w*/*v*, m.wt 40 kD; MP Biomedicals, Illkirch-Graffenstaden, France) in autoclaved drinking water and provided ad libitum [47]. Control (untreated; UT) mice received autoclaved tap water only. The DSS solution was replaced every 2 days for the duration of the experiment. There was no difference in the amount of water consumption by mice between the groups. Mice were sacrificed by cervical dislocation at 5–7 days post-colitis induction.

### 4.3. Treatment Protocols

The nonpeptide Ang 1–7 receptor agonist AVE0991 (Cat # SML2719, Sigma-Aldrich, St Louis, MO, USA) was dissolved in 0.9% saline (vehicle) at 10 mg/mL and stored at −80 °C. Various doses (0.5, 1, 10, 20, 30, and 40 mg/kg) were freshly prepared from the stock each day of the experiment and administered to mice by intra-peritoneal (i.p) injections in a volume of 500 µL per injection. The following protocols (as indicated in the relevant figures) were used: (a) prophylactic approach as single i.p injection on day 1, (b) prophylactic approach as two i.p injections on days 1 and 3, (c) prophylactic approach as daily i.p injections on days 1–5, (d) treatment approach as single injection (at day 5), and (e) treatment approach as daily i.p injections (at days 5, 6, and 7, either as monotherapy or various combination regimens). The other drugs used in the combination regimens were as follows: budesonide (Cat # 01901680540365, AstraZeneca, Cambride, UK), methylprednisolone (Cat # P6004, Sigma-Aldrich, Darmstadt, Germany), and azathioprine (Cat # A4638, Sigma-Aldrich, Darmstadt, Germanyt).

### 4.4. Gross (Macroscopic) Assessment of Colitis Severity

Using sterile forceps and scissors, the entire colon of each mouse was removed by a ventral midline incision and opened longitudinally. Its length and maximal bowel thickness were measured (in mm) with calipers. Several other macroscopic parameters were used to assess the colitis severity, including stool consistency, blood in stool, adhesion, erythema, edema, and anorectal bleeding [47]. The data are presented as the percentage of mice in each group showing these features.

### 4.5. Histological (Microscopic) Assessment of Colitis Severity

The colon was cleaned of stool and blood with a few drops of sterile 0.9% saline and “Swiss-rolled” from the descending to the ascending part. Samples were fixed in 10% neutral buffered formalin and placed in tissue processing and embedding cassettes in PBS for a few minutes and then overnight in a LEICA ASP 3005 tissue processing machine. Tissues were rinsed in two changes of formalin, then dehydrated in several changes of graded alcohol (70%, 90%, and 100%). Three changes of xylene were used for tissue condensation and clearing. Using an SLEE-MPS embedding machine, processed tissues were embedded in paraffin wax (for 24 h at room temperature) and stored at 4 °C before trimming and sectioning using a LEICA RM 2235 microtome. Sections (6 µm thick) were floated in a water bath and then placed on uncoated slides at 37 °C overnight. After de-paraffinization in three changes of xylene (5 min each) and rehydration by serial immersion for 2–3 min in each of absolute, 90%, and 70% alcohol, sections were washed briefly with distilled water and stained in Meyer’s alum hematoxylin solution for 7 min followed by thorough rinsing with running tap water. Before counter-staining sections in eosin solution for 2 min, slides were dehydrated in graded alcohol. A clearing step was performed by rinsing in three changes of xylene (2 min each), followed by mounting with DPX.

The stained sections were (blindly) scored by 3 observers using a standard semi-quantitative histology scoring system [47,48,49], which graded the following features: extent of destruction of normal mucosal architecture (0, normal; 1, 2, and 3, mild, moderate, and extensive damage, respectively), presence and degree of cellular infiltration (0, normal; 1, 2, and 3, mild, moderate, and transmural infiltration, respectively), extent of muscle thickening (0, normal; 1, 2, and 3, mild, moderate, and extensive thickening, respectively), presence or absence of crypt abscesses (0, absent; 1, present), and the presence or absence of goblet cell depletion (0, absent; 1, present). The scores for each feature were summed with a maximum possible score of 11. The extent of ulceration was determined on each section along the muscularis mucosa and expressed as the percentage of ulcerated mucosa [49].

### 4.6. Alcian Blue Stain

Alcian blue staining for the paraffin-embedded colon sections was used to determine the effect of various treatment approaches on the expression level of mucins. The sections were immersed in different solutions as follows: distilled water (30 s), 3% acetic acid (3 min), alcian blue solution (30 s), distilled water (2 min), nuclear-fast red solution (5 min), and distilled water (30 s). Signal intensity estimated in defined fields was measured using the ImageJ software package (version # 1.54p).

### 4.7. Immunofluorescence

Colon sections (5 µm) were deparaffinized and rehydrated through a series of washes in graded ethanol and water, followed by an antigen retrieval step (by boiling the sections in 10 mM sodium citrate buffer, pH 6.0 for 20 min). Sections were then incubated in blocking solution (5% bovine serum albumin (BSA) + 0.3% Triton X-100 in PBS) for 1 h, followed by incubation overnight at 4 °C with primary antibodies [focal adhesion kinase FAK (D5O7U) XP^®^ Rabbit mAb, Cat # 71433, and junctional adhesion molecule JAM-A (E6Z7E) Rabbit mAb, Cat # 82196, (1:50 dilution), from Cell Signaling, Boston, USA]. On the following day, sections were washed and incubated with secondary antibody conjugated to Alexa Fluor 555 (Goat anti-rabbit SFX kit; Life Technologies, USA, 1:400 dilution) for 2 h at room temperature in the dark. After PBS washes, sections were stained with 4′,6 diamidino-2-phenylindole and mounted. Images were captured on a ZEISS LSM 700 confocal microscope, and fluorescence intensity estimated in defined fields was measured using the ImageJ software package.

### 4.8. Western Blotting

Colon tissue samples (descending part) were cut and homogenized (with Teflon glass homogenizer) in 1 mL of buffer composed of 1.2 g of 50 mM HEPES, 0.3 g of 50 mM NaCl, 1 mL of 0.5 M EDTA, 1 mL of 1% Triton X-100, and 98 mL of deionized water. A protease inhibitor cocktail (10 µg/mL aprotinin, 10 µg/mL leupeptin, and 100 µM PMSF) was added separately. Homogenates were centrifuged at 1800 rpm for 10 min at 4 °C, and the supernatant was collected. Protein concentration was determined by the Bradford assay (Bio-Rad, Hercules, CA, USA). Samples containing 50 µg protein were dissolved in an equal volume of 2 x Lammeli sample buffer and β-mercaptoethanol, heated at 90 °C for 10 min, and loaded onto a 12.5% SDS-polyacrylamide gel and electrophoresed at 125 V for 1 h. Proteins were transferred (at 100 V for 1 h) onto a PVDF membrane (Millipore, Cork, Ireland) and then blocked with 4% BSA for 90 min before overnight incubation at 4 °C with primary antibodies for actin (Cat # 4967), phospho-ERK1/2 (Cat # 9102), phospho-p38 MAPK (Cat # 9212), and phospho-Akt (Cat # 4060) (all from Cell Signaling Technology, Boston, MA, USA; 1/1000 dilution). Membranes were washed 3 times for 1 h with 1x TBS-T buffer and incubated with appropriate horseradish peroxidase (HRP)-labeled secondary antibodies [Anti-rabbit IgG, HRP linked antibody (Cell Signaling Technology, Boston, MA, USA; 1/1000 dilution), and Donkey anti-goat IgG-HRP linked antibody (Santa Cruz, TX, USA; 1/100 dilution)].

### 4.9. Proteomic Profiling for Colon Homogenates

Colon tissue (100 mg/mL) was homogenized as described above and sonicated for 1 min for further disruption of the cell membrane. Homogenates were centrifuged for 15 min at 5000 rpm, and the supernatants were collected for subsequent analysis. The protein concentration in the samples was determined by the Bradford assay. The relative changes of different cytokines and chemokines were detected using the Proteome Profiler^™^ Mouse Cytokine Array Kit (Catalog # ARY006, R&D Systems, Inc., Minneapolis, MN, USA). The procedure was performed according to the manufacturer’s protocol. In brief, nitrocellulose membrane containing duplicate spots of selected capture antibodies was incubated in 2 mL of Array Buffer 6 (works as blocking buffer) in 4-well multi-dish on a rocking platform for 1 h. For each membrane, colonic homogenate was diluted with 500 μL of Array Buffer 4 and 940 μL of Array Buffer 6, mixed with 15 μL of reconstituted cocktail of biotinylated detection antibodies, and incubated overnight with the array after aspirating Array Buffer 6 from the wells. The cytokine and detection antibody complex was bound to its conjugate antibody, which was located and immobilized on the membrane. After the incubation, 3 times washing with 1× washing buffer each for 10 min was carried out for each membrane separately. Diluted streptavidin-HRP was then added to each membrane in the wells and incubated for 30 min. Chemiluminescent detection reagents were added to the membranes after 3 washes with the washing buffer, and a signal was produced relative to the amount of bound cytokine. The spot intensity of each protein was quantified using the GS 800 calibrated densitometer (Quantity One software version 4.6.9, Bio-rad^®^), and the average of duplicate spots on each membrane was normalized with the average control spots provided for each membrane [50].

### 4.10. Statistical Analysis

Data were analyzed using GraphPad Prism version 5.0 for Windows (GraphPad Software, Boston, MA, USA). Differences between groups were assessed using one-way ANOVA followed by the Bonferroni post hoc test, with *p* ≤ 0.05 regarded as statistically significant.

## Figures and Tables

**Table 1:** Effect of administration of a single or double dose of AVE0991 on the macroscopic parameters of colitis severity using the prophylactic approach.

Group	Edema	Erythema	Diarrhea	Blood in Stool	Anorectal Bleeding	Adhesions
UT	0	0	0	0	0	0
Vehicle	60 *	0	66 *	66 *	0	66 *
1 mg/kg Single dose	0 #	0	0 #	0 #	0	0 #
1 mg/kg Double dose	0 #	0	0 #	0 #	0	0 #
20 mg/kg Single dose	0 #	0	0 #	0 #	0	0 #
20 mg/kg Double dose	0 #	0	0 #	0 #	0	0 #

Colitis severity (reflected by indicated parameters) was assessed in untreated (UT) mice or in mice receiving i.p AVE0991 along with DSS treatment. * denotes a significant difference from UT mice; # denotes a significant difference from DSS/i.p saline-treated mice, with *p* < 0.05.

**Table 2:** Effect of daily administration of AVE0991 on the macroscopic parameters of colitis severity using the prophylactic approach.

Group	Edema	Erythema	Diarrhea	Blood in Stool	Anorectal Bleeding	Adhesions
UT	0	0	0	0	0	0
Vehicle	100 *	0	66 *	66 *	0	100 *
1 mg/kg	33 #	0	0 #	0 #	0	0 #
20 mg/kg	0 #	0	0 #	0 #	0	0 #
40 mg/kg	0 #	0	0 #	0 #	0	0 #

Colitis severity was assessed in UT mice or in mice receiving i.p AVE0991 along with DSS treatment. * denotes a significant difference from UT mice; # denotes a significant difference from DSS/i.p saline-treated mice, with *p* < 0.05.

**Table 3:** Effect of a single dose administration of AVE0991 on the macroscopic parameters of colitis severity using the treatment approach.

Group	Edema	Erythema	Diarrhea	Blood in Stool	Anorectal Bleeding	Adhesions
UT	0	0	0	0	0	0
Vehicle	0	0	66 *	60 *	100 *	60 *
20 mg/kg single dose	0	0	66 *	30 *	100 *	100 *
40 mg/kg singe dose	0	0	0 #	0 #	100 *	100 *

Colitis severity was assessed in UT mice or in mice receiving i.p AVE0991 along with DSS treatment. * denotes a significant difference from UT mice; # denotes a significant difference from DSS/i.p saline-treated mice, with *p* < 0.05.

**Table 4:** Effect of daily administration of AVE0991 on the macroscopic parameters of colitis severity using the treatment approach.

Group	Edema	Erythema	Diarrhea	Blood in Stool	Anorectal Bleeding	Adhesions
UT	0	0	0	0	0	0
Vehicle	100 *	100 *	100 *	100 *	100 *	100 *
0.5 mg/kg	33 #	33 #	100 *	100 *	100 *	100 *
1 mg/kg	33 #	0 #	100 *	100 *	100 *	100 *
10 mg/kg	33 #	0 #	100 *	100 *	100 *	33 #
20 mg/kg	33 #	0 #	100 *	100 *	100 *	33 #
30 mg/kg	33 #	0 #	0 #	0 #	0 #	0 #
40 mg/kg	0 #	0 #	0 #	0 #	0 #	0 #

Colitis severity was assessed in UT mice or in mice receiving i.p AVE0991 along with DSS treatment. * denotes a significant difference from UT mice; # denotes a significant difference from DSS/i.p saline-treated mice, with *p* < 0.05.

**Table 5:** Effect of daily administration of azathioprine on the macroscopic parameters of colitis severity using the treatment approach.

Group	Edema	Erythema	Diarrhea	Blood in Stool	Anorectal Bleeding	Adhesions
UT	0	0	0	0	0	0
Vehicle	100 *	100 *	100 *	100 *	100 *	100 *
1 mg/kg	100 *	0 #	25 #	0 #	50 *#	100 *
3 mg/kg	100 *	0 #	25 #	0 #	25 #	100 *
5 mg/kg	100 *	0 #	25 #	0 #	0 #	25 #
10 mg/kg	100 *	0 #	0 #	0 #	0 #	0 #

Colitis severity was assessed in UT mice or in mice receiving azathioprine along with DSS treatment. * denotes a significant difference from UT mice; # denotes a significant difference from DSS/i.p saline-treated mice, with *p* < 0.05.

**Table 6:** Effect of daily administration of AVE0991 (low dose), methylprednisolone, budesonide, or the combination regimen on the macroscopic parameters of colitis severity using the treatment approach.

Group	Edema	Erythema	Diarrhea	Blood in Stool	Anorectal Bleeding	Adhesions
UT	0	0	0	0	0	0
Vehicle	100 *	100 *	100 *	100 *	100 *	100 *
AVE (1 mg/kg)	100 *	0 #	33 #	0 #	33 #	66 *#
MP (5 mg/kg)	33 #	0 #	0 #	0 #	0 #	66 *#
Bud (0.5 µg/kg)	0 #	0 #	0 #	33 #	33 #	33 #
AZA (1 mg/kg)	100	0 #	25 #	0 #	50 #	100
AVE + MP	0 #	0 #	33 #	0 #	0 #	0 #
AVE + Bud	0 #	0 #	0 #	33 #	0 #	0 #
AVE + AZA	0 #	0 #	25 #	0 #	0 #	0 #

Colitis severity was assessed in UT mice or in mice receiving different treatment approaches along with DSS treatment. * denotes a significant difference from UT mice; # denotes a significant difference from DSS/i.p saline-treated mice, with *p* < 0.05.

**Table 7:** Effect of daily administration of AVE0991 (high dose), methylprednisolone, budesonide, or the combination regimen on the macroscopic parameters of colitis severity using the treatment approach.

Group	Edema	Erythema	Diarrhea	Blood in Stool	Anorectal Bleeding	Adhesions
UT	0	0	0	0	0	0
Vehicle	100 *	100 *	100 *	100 *	100 *	100 *
AVE (30 mg/kg)	100 *	0 #	25 #	0 #	25 #	100 *
AZA (1 mg/kg)	100 *	0 #	25 #	0 #	50*#	100 *
MP (5 mg/kg)	33 #	0 #	0 #	0 #	0 #	66 *#
Bud (0.5 µg/kg)	0 #	0 #	0 #	33 #	33 #	33 #
AVE + AZA	0 #	0 #	0 #	0 #	0 #	0 #
AVE + MP	0 #	0 #	0 #	0 #	0 #	0 #
AVE + Bud	0 #	0 #	0 #	0 #	0 #	0 #

Colitis severity was assessed in UT mice or in mice receiving different treatment approaches along with DSS treatment. * denotes a significant difference from UT mice; # denotes a significant difference from DSS/i.p saline-treated mice, with *p* < 0.05.

## Data Availability

All data generated or analyzed during this study are included in this published article.
